# Impact of a Warning CPOE System on the Inappropriate Pill Splitting of Prescribed Medications in Outpatients

**DOI:** 10.1371/journal.pone.0114359

**Published:** 2014-12-05

**Authors:** Chia-Chen Hsu, Chia-Yu Chou, Chia-Lin Chou, Chin-Chin Ho, Tzeng-Ji Chen, Shu-Chiung Chiang, Min-Shan Wu, Sen-Wen Wang, Chung-Yuan Lee, Yueh-Ching Chou

**Affiliations:** 1 Department of Pharmacy, Taipei Veterans General Hospital, Taipei, Taiwan; 2 Department of Critical Care Medicine, Taipei Veterans General Hospital, Taipei, Taiwan; 3 Department and Institute of Pharmacology, National Yang-Ming University, Taipei, Taiwan; 4 Department of Internal Medicine, School of Medicine, National Defense Medical Center, Taipei, Taiwan; 5 Department of Medicine, Tzu Chi University, Hualian, Taiwan; 6 College of Pharmacy, Taipei Medical University, Taipei, Taiwan; 7 Institute of Hospital and Health Care Administration, School of Medicine, National Yang-Ming University, Taipei, Taiwan; 8 Department of Family Medicine, Taipei Veterans General Hospital, Taipei, Taiwan; 9 Information Management Office, Taipei Veterans General Hospital, Taipei, Taiwan; University of Groningen, University Medical Center Groningen, Netherlands

## Abstract

**Background:**

Prescribing inappropriate pill splitting is not rare in clinical practice. To reduce inappropriate pill splitting, we developed an automatic warning system linked to a computerized physician order entry (CPOE) system for special oral formulation drugs in outpatient settings. We examined the impact of the warning system on inappropriate prescribing of pill splitting and assess prescribers' responses to the warnings.

**Methods:**

Drugs with extended-release or enteric-coated formulations that were not originally intended to be split were recognized as “special oral formulations”. A hard-stop system which could examine non-integer doses of drugs with special oral formulations, provide warnings to interrupt inappropriate prescriptions was integrated in CPOE in a medical center since June 2010. We designed an intervention study to compare the inappropriate splitting before and after the implementation of the warning system (baseline period 2010 January to May vs. intervention period 2010 June to 2011 August). During the intervention period, prescription changes in response to a warning were logged and analyzed.

**Results:**

A total of 470,611 prescribed drug items with 34 different drugs with special oral formulations were prescribed in the study period. During the 15-month intervention period, 909 warnings for 26 different drugs were triggered among 354,523 prescribed drug items with special oral formulations. The warning rate of inappropriate splitting in the late intervention period was lower than those in baseline period (0.16% vs. 0.61%, incidence rate ratio 0.27, 95% CI 0.23–0.31, P<0.001). In respond to warnings, physicians had to make adjustments, of which the majority was changing to an unsplit pill (72.9%).

**Conclusions:**

The interruptive warning system could avoid the prescriptions with inappropriate pill splitting. Accordingly, physicians changed their behavior of prescribing special oral formulations regarding inappropriate pill splitting. We suggest the establishment of such system to target special oral formulations with warnings to prevent inappropriate pill splitting.

## Introduction

Special oral formulations, such as extended-release (ER) or enteric-coated (EC) formulations, are an important technology for achieving specific pharmacokinetic profiles [1]. Special oral formulations are usually unsuitable for splitting. Splitting of ER tablets may cause dose dumping and lead to dose-dependent side effects. One adverse drug event (ADE) case was reported in which a crushed extended-release nifedipine tablet resulted in a fatal outcome [2]. As noted in our previously published study, 1% of drugs with special oral formulations (ER or EC tablets not allowed to split) were inappropriately prescribed as split pills at ambulatory settings [3]. Despite full prescribing information on pill splitting in printed hospital formularies, inappropriate pill splitting is still not rare in clinical practice, which threatens patient safety.

Medication errors that lead to ADEs, which frequently occur during prescribing, are the most common cause of harm in outpatient settings and are often preventable [4], [5]. Recent studies suggest that a computerized clinical decision support system (CDSS) in computerized physician order entry (CPOE) system would be effective in preventing the prescribing errors [6]–[9]. However, little data is available regarding the impact of CDSS on the inappropriate pill splitting.

To reduce prescribing inappropriate pill splitting, in 2010, an automatic warning system at outpatient settings was developed in our hospital. In this study, we examined the impact of the warning system on the inappropriate prescribing of pill splitting in our ambulatory CPOE, and assessed the physicians' responses to the warnings.

## Materials and Methods

### Ethics Statement

This study was approved by the institutional review board of Taipei Veterans General Hospital (2012-09-026B). Since the research posed no more than minimal risk to the participants and involved no procedures, the review board agreed that written consent from patients was not required.

### Setting

This study was conducted in an academic medical center which serves more than 2.5 million ambulatory visits per year in Taiwan. In average, about 8,000 prescriptions with 25,000 prescribed drug items were generated for ambulatory patients. All prescriptions were issued by physicians through CPOE that operated since 1993. This integrated CPOE system could perform dosage limit checks, drug duplication checks, drug-drug interaction checks, etc. Since June 2010, a specific interruptive warning system designed by a team of physicians, pharmacists and computer programmers, was integrated into CPOE for blocking prescriptions involving inappropriate pill splitting. We designed an intervention study to compare the inappropriate pill splitting before and after the implementation of the warning system. Baseline period was the 5 months before system implementation from January 1 to May 31 in 2010, and the intervention period was the 15 months after system implementation from June 1 in 2010 to August 31 in 2011.

### Drugs with Special Oral Formulations

Drugs were recognized as “special oral formulations” if they were ER or EC formulations that were not originally intended to be split. The instruction leaflets accompanying the medicines documented drug formulation and indivisibility. Inappropriate prescribed drug items of pill splitting were defined as the drugs with special oral formulations were fragmented.

### Prescribing Warnings

When prescribers submitted a non-integer quantity drug with special oral formulation, a real-time warning would pop up on the CPOE screen. The content of the pop-up warning included the drug's name and a short warning message: “Drug should not be split. Please revise the prescription.” Prescribers had to adjust the drug regimen or change to another medication in response to these warnings in order to complete the prescription process. No further information would be provided after these warnings, such as alternatives or other educational information. However, prescribers could find drug information and lists of alternatives from other online resources.

### Data Collection

Every prescription with special oral formulation drugs during the study period was logged from our ambulatory CPOE system. The measure of analysis was individual prescribed drug item. During intervention period, all prescribed drug items with warnings were collected and regarded as inappropriate drug items of pill splitting. In the baseline period, inappropriate drug items of pill splitting were retrieved retrospectively by applying the same algorithm of the real warning system adopted in intervention period.

The warning rates of inappropriate splitting were calculated monthly. To minimize the effect of monthly order volume fluctuations and medical staff turnover, each 5-month period was defined as below for further statistical analysis: baseline period (5 months before intervention, from January 1 to May 31 in 2010); period 1 (the 1^st^–5^th^ months after intervention, from June 1 to October 31 in 2010), period 2 (the 6^th^–10^th^ months after intervention, from November 1 in 2010 to March 31 in 2011); and period 3 (the 11^th^–15^th^ months after intervention, from April 1 to August 31 in 2011). We assumed that the warning rate would be stable after 1 year of system implementation, thus we performed the data up to the 15^th^ month (period 3).

Any individual changes of prescriptions in respond to the warnings were confirmed by comparing interrupted prescriptions logged, final prescriptions, and medication history in our CPOE. At the time points of the first, second, twelfth and fifteenth months after intervention were analyzed.

### Data Analysis

All data were linked by the SQL server 2008 (Microsoft Corp., Redmond, WA) and analyzed using the SAS software 9.1 (SAS Institute, Cary, NC, USA). Descriptive statistics was used to summarize prescribed drug items characteristics. The warning rates of prescribed drug items with inappropriate splitting before and after this warning system were compared using Poisson regression. Results were considered statistically significant at P<0.05.

## Results

Thirty-four different drugs with special oral formulations in this study were shown in Support Information (Table S1). A total of 116,088 drug items with special oral formulations were prescribed during the 5-month baseline period, while 354,523 drug items with special oral formulations were prescribed during the 15-month intervention period. The detailed information on all inappropriate prescribed items involving special oral formulation drugs was shown in Table 1. During the intervention period, 909 prescribed drug items with warnings for 26 different drugs were detected, and 93.9% (854/909) were prescribed for adults. Of these drug items which triggered warnings, 24.6% (224/909) were prescribed by cardiologists, 13.8% (125/909) by psychiatrists and 11.9% (108/909) by endocrinologists. Central nervous system agents (45.7%, 415/909) and cardiovascular agents (38.6%, 351/909) showed the most frequent warnings. The top three drugs which most frequently triggered warnings were alprazolam ER tab 0.5 mg (22.2%, 202/909), fluvastatin ER tab 80 mg (18.8%, 171/909) and paliperidone ER tab 3 mg (6.9%, 63/909).

**Table 1:** Characteristics of ambulatory prescribed drug items with oral extended-release or enteric-coated formulations that should not be split over the study period.

	Baseline Period		Intervention Period	
	(January to May, 2010)		(June, 2010 to August, 2011)	
**Total prescription number**	**703**	**100%**	**909**	**100%**
**Patient age**				
<18 yrs	14	2.0%	55	6.1%
18–64 yrs	238	33.9%	329	36.2%
≧65 yrs	451	64.2%	525	57.8%
**Patient gender**				
Male	364	51.8%	428	47.1%
Female	339	48.2%	481	52.9%
**Prescriber specialty**				
Cardiology	188	26.7%	224	24.6%
Psychiatry	87	12.4%	125	13.8%
Metabolism & endocrinology	137	19.5%	108	11.9%
Neurology	85	12.1%	98	10.8%
Surgery	41	5.8%	76	8.4%
Nephrology	45	6.4%	52	5.7%
General medicine	47	6.7%	35	3.9%
Others	73	10.4%	191	21.0%
**Top 10 drugs** a **, by therapeutic class** b				
Central Nervous System Agents	211	30.0%	415	45.7%
Alprazolam XR tab 0.5 mg (Xanax)	117	16.6%	202	22.2%
Paliperidone ER tab 3 mg (Invega)	9	1.3%	63	6.9%
Bupropion SR tab 150 mg (Wellbutrin)	60	8.5%	50	5.5%
Valproate EC tab 200 mg (Depakine)	18	2.6%	42	4.6%
Diclofenac SR tab 75 mg (Meitifen)	1	0.1%	29	3.2%
Others	6	0.9%	29	3.2%
Cardiovascular Agents	369	52.5%	351	38.6%
Fluvastatin XL tab 80 mg (Lescol)	224	31.9%	171	18.8%
Felodipine ER 5 mg (Plendil)	48	6.8%	52	5.7%
Doxazosin XL tab 4 mg (Doxaben)	27	3.8%	38	4.2%
Others	70	10.0%	90	9.9%
Anti-Diabetic Agents	74	10.5%	59	6.5%
Metformin ER tab 500 mg (Ansures)	74	10.5%	59	6.5%
Enzymes	10	1.4%	31	3.4%
Serratiopeptidase tab 5 mg (Danzen)	10	1.4%	31	3.4%
Others	39	5.5%	53	5.8%

a Top 10 drugs which most frequently triggered warnings during the intervention period.

b Drugs were classified by the American Hospital Formulary Service (AHFS) Pharmacologic-Therapeutic Classification System.

**Figure 1:**
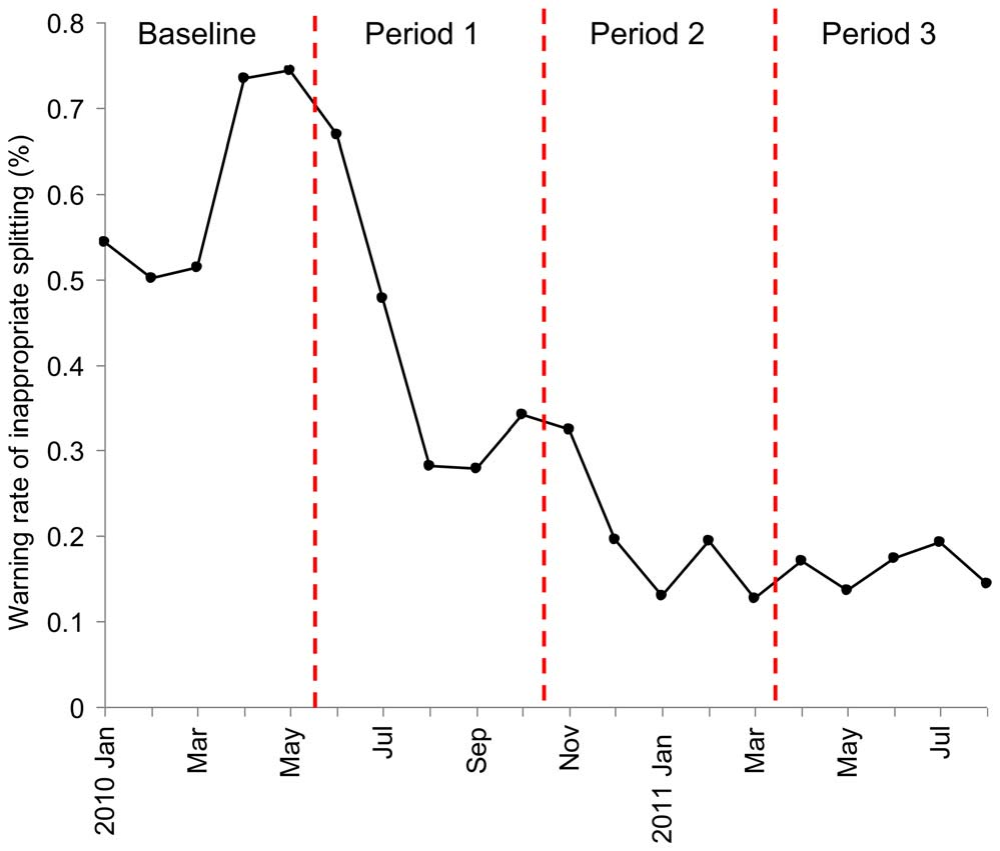
Monthly weaning rate of inappropriate pill splitting during 5-month baseline period and 15-month intervention period. The implementation date of the warning system: June 1, 2010. Baseline period was the 5 months before intervention (from January 1 to May 31 in 2010); period 1 was the 1^st^–5^th^ months after intervention (from June 1 to October 31 in 2010); period 2 was the 6^th^–10^th^ months after intervention (from November 1 in 2010 to March 31 in 2011); period 3 was the 11^th^–15^th^ months after intervention (from April 1 to August 31 in 2011). A significant reduction of weaning rate of prescriptions containing inappropriate splitting was found in period 1 to 3, as compared to the baseline period (p<0.001).

The inappropriate drug items associated with a warning had a rapid and sustained decrease after the implementation of the system (Figure 1). In the baseline period, the warning rate of inappropriate drug items was 0.61% (703/116,088), and then the rate dropped to 0.41% (488/117,907) in period 1, 0.19% (235/121,979) in period 2, and 0.16% (186/114,637) in period 3. Compared with the baseline period, the incidence rate ratio (IRR) of warning rate was gradually reduced from period 1 (IRR 0.68, 95% CI 0.61–0.77, P<0.001), period 2 (IRR 0.32, 95% CI 0.27–0.37, P<0.001) to period 3 (IRR 0.27, 95% CI 0.23–0.31, P<0.001). The warning rate of inappropriate prescribing in period 2 decreased significantly (IRR 0.47, 95% CI 0.40–0.54, P<0.001), compared to period 1. However, there was no significant decrease from period 2 to period 3 (IRR 0.84, 95% CI 0.69–1.02, P = 0.08).

When compared to the baseline period, the warning rate of inappropriate pill splitting at period 3 showed a significant reduction in prescribed drug items for adult patients (Table 2). The warning rate also significantly reduced by the end of the study period in the top 7 specialties (including cardiologists, endocrinologists, psychiatrists, neurologists, general internists, nephrologists, and surgeons) which showed the highest prescribing rate of inappropriate pill splitting in the baseline period. The warning rates of top 10 drugs with the most frequent warnings in the baseline period were also reduced, except for valproate EC tab 200 mg.

**Table 2:** Comparison of warning prescriptions with inappropriate pill splitting in the baseline period and in the third intervention period, stratified by patient age, prescriber specialty and the specific drug.

	Baseline Period			The 3^rd^ Intervention Period			Incidence Rate Ratio	95% CI	P value
	5 months			11^th^–15^th^ month					
	n*	N	(%)	n	N	(%)			
**Patient age**									
<18 yrs	14	2133	(0.66)	13	2028	(0.64)	0.98	0.46–2.08	0.95
18–64 yrs	238	43960	(0.54)	75	47086	(0.16)	0.29	0.23–0.38	<0.001
≧65 yrs	451	69995	(0.64)	98	65523	(0.15)	0.23	0.19–0.29	<0.001
**Prescriber specialty**									
Cardiology	188	21906	(0.86)	42	19206	(0.22)	0.25	0.18–0.36	<0.001
Metabolism & endocrinology	137	8409	(1.63)	27	7677	(0.35)	0.22	0.14–0.33	<0.001
Psychiatry	87	6983	(1.25)	27	7568	(0.36)	0.29	0.19–0.44	<0.001
Neurology	85	10114	(0.84)	13	9801	(0.13)	0.16	0.09–0.28	<0.001
General medicine	47	6192	(0.76)	7	5110	(0.14)	0.18	0.08–0.40	<0.001
Nephrology	45	5778	(0.78)	5	5889	(0.08)	0.11	0.04–0.27	<0.001
Surgery	41	8305	(0.49)	17	17449	(0.10)	0.20	0.11–0.35	<0.001
Others	73	48401	(0.15)	48	41937	(0.11)	0.76	0.53–1.09	0.14
**Drugs**									
Fluvastatin XL tab 80 mg (Lescol)	224	4242	(5.28)	34	4117	(0.83)	0.16	0.11–0.22	<0.001
Alprazolam XR tab 0.5 mg (Xanax)	117	1793	(6.53)	34	4347	(0.78)	0.12	0.08–0.18	<0.001
Metformin ER tab 500 mg (Ansures)	74	2556	(2.90)	17	2826	(0.60)	0.21	0.12–0.35	<0.001
Bupropion SR tab 150 mg (Wellbutrin)	60	1672	(3.59)	14	1779	(0.79)	0.22	0.12–0.39	<0.001
Felodipine ER 5 mg (Plendil)	48	13446	(0.36)	9	11188	(0.08)	0.23	0.11–0.46	<0.001
Doxazosin XL tab 4 mg (Doxaben)	27	6589	(0.41)	8	6725	(0.12)	0.29	0.13–0.64	0.002
Diltiazem retard tab 90 mg (Cardizem)	26	3730	(0.70)	2	2132	(0.09)	0.13	0.03–0.57	0.006
Valproate EC tab 200 mg (Depakine)	18	2281	(0.79)	8	2108	(0.38)	0.48	0.21–1.11	0.09
Nifedipine OROS tab 30 mg (Adalat)	18	10354	(0.17)	6	9672	(0.06)	0.36	0.14–0.90	0.03
Potassium chloride tab 600 mg (Slow-K)	15	1168	(1.28)	1	1243	(0.08)	0.06	0.01–0.47	0.007
Diphenylmethane EC tab 5 mg (Bisacodyl)	15	9099	(0.16)	2	9423	(0.02)	0.13	0.03–0.56	0.007

n, number of prescriptions with warnings; N, number of prescriptions with special oral formulations; %, n/N, proportion of prescriptions with warnings.

* Inappropriate prescriptions of pill splitting were retrieved retrospectively by applying the same algorithm of the real warning system adopted in intervention period.

A total of 55 drug items with 13 different drugs were prescribed inappropriately for pediatric patients during 15-month intervention period. We found that serratiopeptidase tab 5 mg had a large percentage (50.9%, 28/55) of inappropriate drug items in pediatrics. Loratadine/pseudoephedrine repetabs tab 10/240 mg, valproate EC tab 200 mg, potassium chloride tab 600 mg, and diphenylmethane EC tab 5 mg were observed in 12.7% (n = 7), 9.1% (n = 5), 5.5% (n = 3) and 5.5% (n = 3) of inappropriate items, respectively. As Table 2 showed, we found that the warning rates did not change in prescriptions for pediatrics. Among these 13 different drugs with special oral formulations, 5 different drugs had alternatives with liquid formulation. Another 7 different drugs had immediate-release formulation with same ingredient or efficacy. There was no suitable alternative drug for serratiopeptidase tab.

Four kinds of physicians' responses to the warnings included changing to an unsplit pill by changing dose or frequency (72.9%, 248/340), switching to same-ingredient with different formulation or with the same formulation but lower strength (11.8%, 40/340), switching to different ingredient alternatives (10.3%, 35/340), and cancelling the drug item which triggered the warning (5.0%, 17/340). The physicians' responses of the top 10 most frequently inappropriate prescribed drugs were also analyzed (Table 3). The proportion of waving prescription (50%, 4/8) in serratiopeptidase tab was highest.

**Table 3:** Physicians' responses of the top 10 frequent inappropriate drug prescriptions when receiving hard-stop warnings.^a^

	Total	Change to unsplit pill^b^		Switch to same ingredient product^c^		Switch to another ingredient product^d^		Waive prescription	
**Total**	**340**	**248**	**72.9%**	**40**	**11.8%**	**35**	**10.3%**	**17**	**5.0%**
Alprazolam XR tab 0.5 mg (Xanax)	87	50	57.5%	24	27.6%	11	12.6%	2	2.3%
Fluvastatin XL tab 80 mg (Lescol)	80	67	83.8%	-e		12	15.0%	1	1.3%
Paliperidone ER tab 3 mg (Invega)	7	6	85.7%	-e		1	14.3%	0	
Metformin ER tab 500 mg (Ansures)	26	15	57.7%	7	26.9%	1	3.8%	3	11.5%
Felodipine ER 5 mg (Plendil)	19	8	42.1%	6	31.6%	3	15.8%	2	10.5%
Bupropion SR tab 150 mg (Wellbutrin)	24	20	83.3%	-e		4	16.7%	0	
Valproate EC tab 200 mg (Depakine)	13	11	84.6%	2	15.4%	0		0	
Doxazosin XL tab 4 mg (Doxaben)	16	8	50.0%	5	31.3%	2	12.5%	1	6.3%
Serratiopeptidase tab 5 mg (Danzen)	8	4	50.0%	-e		-e		4	50.0%
Diclofenac SR tab 75 mg (Meitifen)	3	3	100.0%	0		0		0	
Others	57	50	87.7%	2	3.5%	1	1.8%	4	7.0%

a At the points of the first (June, 2010), second (July, 2010), twelfth (May, 2011) and fifteenth (August, 2011) months after intervention.

b For example, 0.5 tab twice daily changed to 1 tab once daily; 0.5 tab once daily changed to 1 tab once daily; or 1.5 tab once daily changed to 1 tab once daily.

c Products with different formulations or with the same formulation but lower strength.

d Products with the same therapeutic effects.

e No available products in the study hospital.

## Discussion

The interruptive warning system showed clinical significant impact to avoid inappropriate pill splitting. After the system was implemented, the warning rate rapidly declined. In respond to warnings, physicians had to make adjustments. The warnings substantially affected the prescribing behavior in regard to pill splitting of special oral formulations.

The impact of an alert of inappropriate pill splitting in a CDSS has been reported. Quinzler et al. developed and implemented a CDSS which provided alerts of inappropriate pill splitting during the prescribing process for ambulatory patients or patients at discharge [10]. Their results showed that the CDSS application could significantly reduce the rate of inappropriate splitting. However, our study design differed from theirs. Our studied drugs were special oral formulations that did not allow splitting, while in the study of Quinzler et al., they included all capsules and unscored tablets which were unsuitable to be split whether they were ER/EC formulations or not. Base on pharmacokinetics, inappropriate splitting of extended-release or enteric-coated pills would affect drug efficacy and result in side effects. Therefore, splitting pills with special oral formulations should be forbidden. Recent studies also reported the adverse clinical consequence of tablet crushing [11], [12]. As for the normal capsules and tablets, split pills did not seem to affect the clinical outcomes in patients with hypertension, hyperlipidemia or psychiatric disorders [13]. This was the reason that we designed the warning system only for extended-release or enteric coated pills which did not allow splitting. In addition, our interruptive warning system can completely avoid the inappropriate splitting. Compared to the alert system in the study of Quinzler et al., only nearly half (2.7% decreased to 1.4%) of inappropriate splitting was prevented, which meant that still one half of alerts were overridden by prescribers.

In our study, a clear learning effect was observed on physicians' practice behavior after the implementation of the warning system for prescriptions. After physicians experienced inappropriate splitting warnings, the warning rates showed a remarkable reduction. Inadequate physician knowledge about drug formulations may contribute to the majority of prescribing errors [14], [15]. Most clinicians have not continually received proper pharmacokinetic education regarding the use of drugs with special oral formulations, thereby resulting in prescription errors. We identified this limitation in physician education, so we implemented this warning system to avoid inappropriate pill splitting. Accordingly, prescribers changed their behavior on prescribing drugs with special oral formulations when they received a warning. The remained few warnings in the late intervention period might be due to (1) physicians who had never experienced the warning of a specific drug, or (2) physicians who forgot or neglected the indivisibility of special oral formulations.

We observed that the warning rate of inappropriate pill splitting in pediatric patients seemed not to be affected over the study period. A pediatric dosage must be adjusted by age, body weight and disease conditions. Thus, a lack of child-friendly formulations may result in pill splitting [16]. Even though some pills had alternatives with liquid formulations, physicians still preferred split pills. The reasons might be that liquids were more expensive or had unacceptable tastes [17]. It implied that more child-friendly formulations such as granules or chewable tablets may be introduced by the hospital to meet clinical needs.

In the analysis of the modifications in response to warnings, it was noteworthy that physicians cancelled certain prescriptions instead of shifting to alternatives. One of the possible reasons might be that the warnings forced physicians to reevaluate the necessity of drug use. In this study, serratiopeptidase was the only one with special oral formulations that had no alternative drug among 34 different drugs. When physicians received warnings of split serratiopeptidase, they had to change to unsplit pill or cancel the prescription. Serratiopeptidase is a proteolytic enzyme that had been used for relieving inflammation- and edema-associated conditions such as respiratory congestion, trauma, and infection [18]. However, the recommended dose of serratiopeptidase remains unclear particular to pediatrics [19]. In addition, serratiopeptidase is made as an enteric-coated tablet to avoid inactivation of the enzyme activity by gastric acid. Splitting this tablet damaged the protective coating, which resulted in loss of efficacy. Furthermore, because the existing evidence of clinical efficacy of serratiopeptidase was insufficient [19], the renewal of drug license had been withdrawn by Taiwan Food and Drug Administration in 2011 [20]. Due to limited clinical benefit for patients, it seems rational that physicians gave up prescribing serratiopeptidase.

Although the CPOE with CDSS can reduce prescribing errors [21]; but new type of error can be resulted. For example, Strom et al. showed that hard-stop warnings can delay clinically important treatment for inpatients in need of immediate drug therapy [22]. However, our hard-stop warning system was set up in outpatient care, where fewer patients need urgent drug therapy. Furthermore, the possible unintended consequences of hard-stop warnings may occur which require further research.

Previous studies have indicated that inappropriate pill splitting could increase the risk of adverse events and even lead to treatment failure [2], [23]. The warning in prescribing process has been considered an important strategy to prevent medical harm [24]. Our results offer fundamental insights for warning of inappropriate pill splitting. However, the current study has some limitations. First, this study was carried out in a single hospital. The warning system was implemented in outpatient settings of a medical center where attending physicians prescribed the medications. Hence, the generalization of the results on other settings is unknown. Second, this non-randomization study had potential confounding variables, such as medical staff turnover. Third, because we intercepted all prescribed drug items of inappropriate pill splitting, adverse effects related to pill splitting would never occur. Thus we could not evaluate the true clinical impact of this warning system to patients. Fourth, we could not access the appropriateness of physicians' responses to the warnings because of limited data availability in this retrospective study.

## Conclusions

Prescribing inappropriate pill splitting is not rare in clinical practice. The interruptive warning system avoided the prescriptions with inappropriate pill splitting. Physicians would change their behavior of prescribing drugs with special oral formulations owing to a hard-stop warning or a learning effect from previous warnings regarding inappropriate splitting. Thus, interruptive warning system should be developed and implemented in order to prevent prescribing inappropriate pill splitting.

## Supporting Information

Table S1 Drugs with special oral formulations, selected from TVGH formulary.
(DOCX)
