# Supplementary material for: Impact of a Warning CPOE System on the Inappropriate Pill Splitting of Prescribed Medications in Outpatients
Source: PLoS One. 2014 Dec 5;9(12):e114359. doi: 10.1371/journal.pone.0114359 (PMC4257670; doi:10.1371/journal.pone.0114359)
Supplement: Table S1 — Drugs with special oral formulations, selected from TVGH formulary. (DOCX) [file pone.0114359.s001.docx]

**Support Information**

**Table S1.** Drugs with special oral formulations, selected from TVGH formulary^a^.

| Drug formulation and drug class^b^ | Drug name (product name) | Potential problems of splitting^c^ |
| --- | --- | --- |
| **Extended-release formulations** | | |
| **Anti-Diabetic Agents** | Glipizide SR tab 10 mg (Diabetrol^®^); Metformin ER tab 500 mg (Ansures^®^) | May increase risk of hypoglycemia (e.g. gliclazide MR tab), cause gastric irritation (e.g., metformin ER tab), decrease duration of action |
| **Cardiovascular Agents** |  |  |
| Antilipemic Agents | Bezafibrate retard coated tab 200 mg (Bezalip^®^); Fluvastatin XL tab 80 mg (Lescol^®^) | May decrease lipid-lowering efficacy |
| Calcium Channel Blockers | Diltiazem retard tab 90 mg (Cardizem^®^); Felodipine ER tab 2.5 mg, 5 mg (Plendil^®^); Nifedipine OROS tab 30 mg (Adalat^®^) | May deliver a toxic dose of the active ingredient, increase risk of hypotension, decrease duration of action |
| α-Adrenergic Blocking Agents | Alfuzosin XL tab 10 mg (Xatral^®^); Bunazocin ER tab 3 mg (Detantol^®^); Doxazosin XL tab 4 mg (Doxaben^®^) | May increase risk of dizziness, hypotension and reflex tachycardia, decrease duration of action |
| **Central Nervous System Agents** |  |  |
| Analgesics and Antipyretics: NSAIDs | Diclofenac SR tab 75 mg (Meitifen^®^); Etodolac SR tab 600 mg (Lacoxa^®^) | May deliver a toxic dose of the active ingredient, increase gastric irritation |
| Analgesics and Antipyretics: Opiate Agonists | Morphine SR tab 60 mg (MST^®^) | May increase risk of drowsiness and respiratory depression, decrease duration of action |
| Antidepressants | Bupropion SR tab 150 mg (Wellbutrin^®^) | May increase risk of insomnia, increase risk of hypertension |
| Antipsychotics Agents | Paliperidone ER tab 3 mg (Invega^®^) | May result in increased fluctuations in plasma drug levels, leading to potentially worse adherence and outcome |
| Anorexigenic Agents | Methylphenidate ER tab 18 mg, 27 mg (Concerta^®^) | May increase risk of insomnia, increase risk of hypertension and tachycardia |
| Anxiolytics | Alprazolam XR tab 0.5 mg (Xanax^®^) | May increase risk of sedation, decrease duration of action |
| Anti-Parkinson drugs | L-dopa/Benserazide HBS cap 100/25 mg (Madopar^®^) | May increase risk of nausea, vomiting and excessive motor activity, decrease duration of action |
| **Electrolytic Replacement Preparations** | Potassium chloride tab 600 mg (Slow-K^®^) | May cause gastric irritation |
| **Genitourinary Smooth Muscle Relaxants** | Tolterodine SR cap 4 mg (Detrusitol^®^) | May increase risk of somnolence, flushing, and dry mouth, decrease duration of action |
| **Respiratory Tract Agents** |  |  |
| Antihistamines | Loratadine/Pseudoephedrine repetabs tab 5/120 mg (Clarinase^®^), 10/240 mg (Finska-LP^®^) | May deliver a toxic dose of the active ingredient, increase risk of dry mouth, nervousness, decrease duration of action |
| Mucolytic Agents | Ambroxol SR tab 80 mg (Loxol^®^) | May decrease duration of action |
| **Enteric-coated formulations** | | |
| **Anti-Infective Agents: Antivirals** | Didanosine EC DR cap 250 mg, 400 mg (Videx^®^) | May become inactive in the stomach, increase the risk of digestive intolerance |
| **Central Nervous System Agents: Anticonvulsants** | Valproate EC tab 200 mg (Depakine^®^) | May cause gastric irritation |
| **Enzymes** | Serratiopeptidase tab 5 mg (Danzen^®^) | May become inactive in the stomach |
| **Gastrointestinal Agents** |  |  |
| Laxatives | Diphenylmethane EC tab 5 mg (Bisacodyl^®^) | May cause gastric irritation |
| Proton pump inhibitors | Rabeprazole tab 20 mg (Pariet^®^); Pantoprazole tab 40 mg (Pantoloc^®^) | May become inactive in the stomach |
| Anti-inflammatory agents | Mesalamine tab 400 mg (Asacol^®^) | May fail to reach terminal ileum and colon of action |
| **Immunosuppressants** | Mycophenolic acid tab 180 mg (Myfortic^®^) | May fail to reach small intestine of action, increase risk of gastrointestinal side effects |

Cap, capsule; CR, controlled-release; DR, delayed release; EC, enteric-coated; ER, extended-release; HBS, Hydrodynamically Balanced System; MR, modified release; NSAID, non-steroidal anti-inflammatory and antirheumatic products; OROS, osmotic-controlled release oral delivery system; PR, prolonged release; SR, sustained release; tab, tablet; TVGH, Taipei Veterans General Hospital; XL, extended-release; XR, extended-release.

^a^Taipei Veterans General Hospital Formulary 2011 Edition.

^b^Drugs were classified by the American Hospital Formulary Service (AHFS) Pharmacologic-Therapeutic Classification System.

^c^Pharmacologic and formulation considerations.
